# The Case for the Use of Nurse Practitioners in the Care of Children with Medical Complexity

**DOI:** 10.3390/children4040024

**Published:** 2017-04-07

**Authors:** Cheryl Samuels, Tomika Harris, Traci Gonzales, Ricardo A. Mosquera

**Affiliations:** Department of Pediatrics, McGovern Medical School, University of Texas Medical School, Houston, TX 77030, USA; Tomika.S.Harris@uth.tmc.edu (T.H.); Traci.R.Gonzales@uth.tmc.edu (T.G.); Ricardo.A.Mosquera@uth.tmc.edu (R.A.M.)

**Keywords:** nurse practitioner, children with medical complexity, medical homes

## Abstract

Although children with medically complex illness represent less than one percent of the total pediatric population, their health care expenditures and health care system utilization far exceed the numbers of other pediatric patients. Nurse practitioners, with their educational background focused on health care promotion and education, are uniquely qualified to reduce this inequity with cost effective care. Currently, nurse practitioners are used in a variety of health care settings and can provide acute and chronic care. Incorporating nurse practitioners at each step in the care of children with medical complexity can improve the quality of life for these children and their families, increase family satisfaction and decrease costs.

## 1. Introduction

Children with medical complexity (CMC) are a subgroup of children with chronic illnesses and special health care needs who have complex medical conditions that involve multiple body systems. These patients require care from multiple specialists and rely either periodically or chronically on assistive technological devices [1]. While CMC represent less than 1% of the pediatric population, their health care costs far exceed that of their peers, and they account for nearly one-third of all pediatric health system expenses [2]. This population may benefit from more intensive care coordination and focused attention than children without chronic conditions.

The American Academy of Pediatrics (AAP) recognizes that in order for CMC to receive effective care, it must be easily accessible, cohesive, and coordinated [3]. Unfortunately, there are many barriers that prevent community clinicians from being able to provide this type of care within a medical home model. Some of these barriers include lack of training and knowledge regarding complex children, high patient volumes that result in decreased available time to spend with individual patients, limited resources, inadequate staffing, and poor reimbursement [4,5,6]. Nurse Practitioners, (NP) help overcome these barriers by providing comprehensive, cost effective care in the medical home, specialty clinics, and in hospital settings. Care that includes a NP as part of a multi-disciplinary team has been shown to result in better health outcomes and increased family satisfaction [7,8,9]. Both the International Council of Nurses (ICN) and the National Association of Pediatric Nurse Practitioners (NAPNAP), a U.S. organization, recognize the important role that NPs fill and the importance of CMC receiving care in a medical home that includes NPs as key team members [10,11]. This paper will define the role of the nurse practitioner, discuss how they fit into the multidisciplinary healthcare team, and describe many of the specific roles and functions that they provide to improve outcomes for children with medical complexity. 

## 2. What is a Nurse Practitioner?

A nurse practitioner is a licensed provider who has undergone extensive training that allows them to practice independently in multiple healthcare settings. An NP first starts their professional career as a registered nurse (RN), they then complete an accredited master’s or post master’s nursing degree. After completion of the program, they must pass a certification exam from a national governing body before they are allowed to practice. Some NPs also elect to complete a doctorate level degree. NP programs are designed to not only impart the clinical and professional competency needed to provide care in primary, specialty and acute care settings, but also to “cultivate advanced skills in the roles of educator, counselor, advocate, consultant, manager, researcher, and mentor”. As a part of their education, NPs undergo advanced level training in pharmacology, pathophysiology, physical assessment, and clinical diagnosis. This specialized training allows them to diagnose and treat with both pharmacologic and non-pharmacologic measures independently [12]. Currently, the level of independent practice and prescriptive authority varies between states. 

When compared to nurses prepared at the associate or baccalaureate level, NPs have a higher level of knowledge in both disease processes and management. Their skill set is beyond that of a RN and they are able to independently intervene in a patient’s care when needed. The medical care of CMC is multifaceted, often involving multiple specialists, therapies and treatment regimens. These children require extensive coordination and frequent medical intervention. NPs, with their unique training, can effectively coordinate and provide timely medical intervention to these fragile patients [13]. Care provided by NPs has been proven to be cost-effective, with increased patient satisfaction [14], and results in equal or better outcomes when compared to care provided by physician colleagues in similar roles [1]. Because of their advanced education and knowledge, NPs are able to effectively coordinate care between the primary and the hospital team, specialists, and home care services. NPs can also manage or co-manage the care of CMC during inpatient stays and are actively involved in the discharge process. 

## 3. Care Coordination

NPs can serve as effective care coordinators for CMC. Care coordination is the organization of all aspects of a patient’s care between multiple providers and typically occurs outside of provider visits. Care coordination activities include timely exchange of information with specialists and communication with home health providers and family caregivers. In pediatrics, care coordination includes not only medical needs, but also developmental, educational, dental, social, and financial concerns. Each of these aspects is an integral part to ensuring optimal health and wellness is achieved [1]. Frequent communication with families by the NP can result in proactive—rather than reactive—treatment. Early treatment and management of acute illness can potentially help decrease the seriousness of illness and unnecessary hospitalization [13]. While coordination of care is a critical component necessary for achievement of optimal health in all children, it is inherently more important in CMC due to their large number of medical needs [3]. With the higher level of clinical acuity, NPs are better prepared to provide this level of care in CMC than registered nurses. As described by Lohman’s Value Model, advanced nursing training and skills are best used for coordinating care in complex patients where basic nursing skills can be appropriate for general pediatric patients [13].

Incorporating NPs as the primary source of care coordination has been shown in several trials to result in improved patient outcomes. In one randomized controlled trial (RCT), care coordination provided by NPs resulted in a decrease in the number of unmet needs and improved satisfaction with patient care and health [15]. A more recent RCT by Mosquera et al. demonstrated that with the use of NPs for both care coordination and primary care providers, there was a reduction in the number of emergency room visits and hospital admissions, as well as a reduction in overall health care costs while improving family satisfaction in a medical home model [9]. Similarly, a hospital-based clinic for CMC also improved outcomes including a reduction in hospital length of stay along with high satisfaction and reduced costs [16]. Cady et al. demonstrated a reduction in hospitalizations with children that received telehealth care coordination by NP [1]. 

Another integral part of care coordination for CMC is providing a link between the primary care provider (PCP), and pediatric specialists. CMC often have multi-organ system involvement which requires care by multiple specialists with the average CMC being followed by six different pediatric specialists [2]. The ever-expanding population of CMC has resulted in an increased demand for pediatric subspecialists. Unfortunately, there is a shortage of trained pediatric physician subspecialists in the U.S. [17]. As a result of this shortage, it is often difficult for patients to have access to specialists in a timely manner. Depending on the state and specialty, NPs can see new consults, follow up appointments or acute visits in various specialties.

Incorporating NPs into outpatient specialty clinics that previously used only physician providers would likely allow for increased availability of appointments and increased patient access. Kwon performed a 6-month intervention that involved incorporating NPs more heavily into a subspecialty outpatient clinic. Before the implementation, the clinic was only able to accommodate about 23% of received referrals in a single month. The average wait time for an appointment was 3–4 weeks and patient satisfaction was low. Using an expanded role of the nurse practitioner, the clinic was able to more than double the amount of referrals seen in a month to 62%. Patient satisfaction increased and patients were pleased with the care that the NP provided, which often included longer face-to-face time and more patient education [18]. In addition, incorporating NPs in a complex care clinic allowed for better communication and decreased fragmentation of care between specialists and primary care. Decreasing the provider–patient ratio allowed more time for NPs to interact with specialists, avoiding further fragmentation of care and improving communication between specialists and families [9]. 

Because of the chronic, complex nature of their conditions, CMC are at an increased risk for acute exacerbations of their chronic illnesses that may require hospital admission. Depending on the definition of CMC used, authors have documented that CMC account for 40–66% of pediatric hospitalizations, have a longer mean hospital length of stay, and account for 90% of medical deaths among U.S. children [2,5]. Furthermore, CMC experience an increased rate of readmissions. In a study conducted by Berry, readmission rates in CMC were documented at 21.8%. Even more concerning, a small subset of 2.9% experienced four or more readmissions, and these children accounted for nearly 20% of the total hospital admissions during their trial. Between 3 and 23% of those admissions were considered preventable by study personnel [19,20,21]. As insurance providers and hospitals apply pressure to prevent pediatric readmissions, there are also increasing expectations to discharge patients sooner and increase the cost-effectiveness of care provided in acute care settings [19].

One way to effectively address both of these goals is to incorporate nurse practitioners in both the hospital care for CMC and in the discharge process. Several recent studies showed that when utilized in the acute setting discharge process, NPs were able to make a significant impact. Dunn described a unique role created and assigned to a NP to expedite and improve the discharge process. He/she was responsible for patient education, acting as a liaison between various medical teams and providing care coordination to facilitate the transition to a home setting. A similar role was utilized in a project with a NP as a unit-based discharge coordinator. This integral member of a multidisciplinary team helped improve outcomes by enhancing satisfaction, reducing length of stay, and improving the patient discharge process [16,22,23]. Incorporating NPs into the acute care setting as onsite providers and care coordinators resulted in improved satisfaction among staff, decreased length of stay, and a decrease in overall healthcare cost [24]. NPs can also play a critical role in safe discharges. Safe discharges start at admission with clearly established discharge criteria that ensure that necessary supplies, private duty nursing and medications are obtained in a timely fashion and do not delay discharge [23]. Discharges need to be thorough, complete, and involve families so that unnecessary adverse events do not occur at a time when these already medically fragile patients are more vulnerable [16]. Follow-up phone calls made by the NP allowed reinforcement of discharge instructions and encouraged correct use of medications. Phone calls made within 2–3 days of discharge helped to reduce the readmission rates by 26% [25]. Each of these measures, designed to improve hospital-to-home transition, have been proven to be safely and effectively performed by a NP. 

Another critical transition period for CMC is the move from pediatric to adult clinicians. As technology and medicine continue to improve, the number of CMC surviving into adulthood has increased to 90% [26]. Historically, the transition to adult medicine has been associated with lower levels of patient and family satisfaction, feelings of uncertainty and abandonment, and fragmentation of care [24,25]. Strong communication skills and empathetic listening are required to have a positive impact on the process. Using a NP-led comprehensive model that starts the transition discussions in early adolescence and follows them into adulthood has been shown to improve the transition experience [25].

## 4. Conclusions

Regardless of whether a family’s journey with CMC begins at birth, in the neonatal intensive care unit, or later in childhood following a catastrophic illness or injury, effective care coordination in all aspects of care is imperative to reaching optimal health. Nurse practitioners who specialize in the management of CMC, with their holistic preparation and advanced assessment skills, are integral to care coordination, patient care, and family satisfaction. In primary care offices, specialty clinics and hospital settings, incorporating nurse practitioners in the care of children with medical complexity can decrease costs, increase patient satisfaction, and improve the quality of life for these children and their families.
